# Fatal Case of Stevens-Johnson Syndrome/Toxic Epidermal Necrolysis Induced by Doxycycline or Flucloxacillin in a 77-Year-Old Woman: A Rare but Serious Adverse Drug Reaction

**DOI:** 10.7759/cureus.87202

**Published:** 2025-07-02

**Authors:** Shilpa Johnson, Shahina M Patel, Asuramuni De Silva

**Affiliations:** 1 Internal Medicine, Royal Blackburn Hospital, Blackburn, GBR; 2 General Internal Medicine, Royal Blackburn Hospital, Blackburn, GBR

**Keywords:** drug rash, intravenous immunoglobulins (ivig), stevens-johnson syndrome and toxic epidermal necrolysis (sjs), stevens-johnson-syndrome (sjs), toxic epidermal necrolysis (ten)

## Abstract

Stevens-Johnson syndrome (SJS) is a serious, potentially life-threatening condition affecting the skin and mucous membranes. This syndrome occurs twice as frequently in females compared to males and affects individuals across all age groups. The most common triggers are drugs. Here, we present a case of SJS linked to doxycycline/flucloxacillin-induced SJS.

A 76-year-old woman with Sjögren’s syndrome, rheumatoid arthritis (RA), chronic obstructive pulmonary disease (COPD), and osteoarthritis (OA) was referred by her General Practitioner (GP) with a rapidly spreading, itchy, hyperkeratotic rash involving >80% body surface area (BSA), which began on the lower abdomen two days prior. She had received flucloxacillin, doxycycline, and topical steroids, after which the rash worsened. With no known drug allergies, the medications were discontinued, and she was referred for further care. On examination, she had extensive flaky, eruptive lesions with a positive Nikolsky sign. Skin biopsy and immunohistochemistry confirmed widespread epidermal necrosis. A provisional diagnosis of drug-induced toxic epidermal necrolysis (TEN) was made, with flucloxacillin or doxycycline suspected as causative agents. She was started on IV teicoplanin and clindamycin, and received methylprednisolone for three days, followed by intravenous immunoglobulin (IVIG). Dermatology was consulted, and daily swabs and labs were conducted to monitor for infection. Despite intensive care and an initial improvement in Severity-of-Illness Score for Toxic Epidermal Necrolysis (SCORTEN), her condition deteriorated, and she ultimately succumbed to progressive TEN. This case aims to comprehensively document the clinical progression and therapeutic interventions in a fatal presentation of suspected drug-induced TEN. Management of SJS typically involves discontinuation of the likely drug, hospitalization, and supportive care. Though prompt drug withdrawal was implemented, the patient's condition continued to deteriorate, underscoring the severity of the syndrome. This case highlights a rare instance of doxycycline and flucloxacillin-induced SJS in the elderly. It is crucial to identify and recognize this uncommon but potentially life-threatening adverse reaction to doxycycline and flucloxacillin in general practice when prescribed to elderly individuals.

## Introduction

Stevens-Johnson syndrome (SJS) and toxic epidermal necrolysis (TEN) are serious skin conditions marked by widespread epidermal necrosis and detachment. Over 90% of patients experience mucous membrane involvement, typically affecting two or more separate sites. These exist along a continuum, classified based on the percentage of body surface area (BSA) affected. SJS involves less than 10% BSA detachment, SJS/TEN overlap is marked by 10-30% detachment, and TEN occurs when more than 30% of the BSA is affected [1].

Doxycycline is a broad-spectrum, water-soluble tetracycline antibiotic effective against gram-positive and gram-negative organisms. It is used to treat infections such as acne, malaria, STDs (e.g., chlamydia, syphilis), Lyme disease, and bacterial outbreaks like cholera and typhus. Beyond its antimicrobial action, it exerts anti-inflammatory and immunomodulatory effects. Its high lipophilicity enables deep tissue penetration. Though generally well-tolerated, doxycycline may rarely cause serious adverse reactions such as anaphylaxis, pneumonitis, SJS, or drug-induced liver injury (hepatocellular, cholestatic, or mixed patterns). Flucloxacillin is a beta-lactam penicillin antibiotic mainly effective against gram-positive bacteria, with limited gram-negative coverage. It acts by inhibiting bacterial cell wall synthesis via penicillin-binding proteins, causing cell lysis. Its stability against beta-lactamases, including penicillinases and ESBLs, enhances its therapeutic role. Common adverse effects involve cutaneous hypersensitivity reactions. These range from morbilliform rashes to severe forms like erythema multiforme (EM) and SJS (when mucosa is involved).

SJS and TEN are uncommon conditions, with an estimated incidence of five to six cases per million annually. These conditions are more frequently observed in females, with a female-to-male ratio of roughly 2:1 [2]. Risk factors include the following: HIV infection: individuals with HIV face a greatly increased risk of SJS/TEN, with European studies reporting a 7-9% prevalence (a 12-fold risk) due to immune dysregulation and high-risk drugs like nevirapine and co-trimoxazole [3]; connective tissue disease: people with connective tissue disorders have twice the risk of SJS/TEN, though this figure may be overestimated because acute cutaneous lupus erythematosus can mimic TEN; cancer: patients with cancer exhibit a 30-60-fold higher annual incidence of SJS/TEN compared to the general population. Recent cancer diagnoses and radiation therapy are key factors, with common triggers including trimethoprim-sulfamethoxazole and phenytoin [4]; older adults: the incidence of SJS/TEN rises with age, hitting about 13.7 cases per million in those aged 65 and above, compared to 4.1 per million in individuals under 20 and 3.9 per million in those aged 20-64 [5].

A comprehensive survival analysis from the Registry of Severe Cutaneous Adverse Reactions (RegiSCAR), which included 460 patients, found that the overall acute-phase mortality for SJS/TEN was 23%, with rates ranging from 12% to 49% depending on severity. One-year mortality increased to 34%, likely reflecting the influence of severe comorbidities and older age. In the pediatric population, mortality was observed at 0% for SJS, 4% for SJS/TEN overlap, and 16% for TEN [6].

Although many drugs can trigger SJS/TEN, most cases are due to high-risk medications, such as allopurinol, lamotrigine, aromatic anticonvulsants, antibacterial sulfonamides, and certain NSAIDS (especially oxicam derivatives and cyclooxygenase-2 (COX-2) inhibitors). New anticancer agents like immune checkpoint inhibitors (e.g., ipilimumab, pembrolizumab, nivolumab, atezolizumab) have also been linked, with a meta-analysis showing a fourfold increased risk, reactions starting around 26 days after treatment, and a 37% overall mortality rate [7]. Some cases associated with immune checkpoint inhibitors may actually be SJS/TEN-like reactions-severe immune-mediated bullous eruptions due to enhanced ICI cytotoxicity-rather than true SJS/TEN. Rare reports have linked SJS/TEN to chemical exposures, complementary medicines, vaccinations, and certain foods. In at least 15% of cases, no definitive trigger is identified, leaving open the possibility of hidden exposures from alternative sources or unidentified infections [8].

SJS and TEN are driven by a drug-specific T cell immune reaction. The process begins when the human leukocyte antigen (HLA), a triggering drug, and the T cell receptor (TCR) form a synapse that activates drug-specific CD8⁺ T cells, which then release cytotoxic mediators, leading to keratinocyte apoptosis and epidermal necrolysis.

Genetic predispositions, such as specific HLA polymorphisms, cytochrome P450 2C9 variants, and slow acetylator genotypes, increase susceptibility by affecting drug clearance. Additional contributions come from adenosine triphosphate (ATP)-binding cassette transporters and proteasome components. Keratinocytes also play an active role through HLA-mediated antigen presentation, while neutrophils and alarmins from the innate immune system amplify inflammation. Key mediators include granulysin, Fas ligand, perforin/granzyme, and TNF-α [9]. Cytokines, such as TNF-α and interleukin-15, which foster sustained cytotoxic responses, are linked to increased disease severity and mortality.

SJS, though uncommon, poses a serious risk with certain antibiotics such as doxycycline and flucloxacillin. Older adults are particularly vulnerable due to changes in drug handling, multiple concurrent medications, and weakened immunity. This case highlights the critical importance of cautious antibiotic prescribing, close surveillance, informed patients, and collaborative care to ensure swift identification and response.

## Case presentation

SJS usually presents in different stages. Prodromal symptoms, such as malaise, fever, myalgia, sore throat, and conjunctivitis, can occur before or with the onset of mucocutaneous lesions. Initially, an exanthem-like rash appears as merging, ill-defined red macules on the face and chest, which evolve within one to two days into dusky erythema, purpuric spots, atypical target lesions, and flaccid bullae. As the condition worsens, extensive sheet-like skin detachment and erosions develop (with a positive Nikolsky sign); typically, the scalp is spared while the palms and soles become painfully swollen and red. The acute phase lasts about seven to nine days, followed by re-epithelialization over seven to 21 days, during which patients are at risk for fluid and electrolyte imbalances, sepsis, hypothermia, organ failure, and death from skin failure [10]. All mucosal surfaces can be involved during the acute phase. Typically, the buccal, oro/nasopharyngeal, and anogenital areas are affected, with 80% of patients showing involvement at two or more sites. The oral cavity is most commonly affected (90%), followed by the nasal and ear mucosa (50% each) and the larynx (30%) [11]. Genital involvement occurs in 60-70% of patients, often as erosions and blisters; in women, vulvovaginal lesions (erosive vaginitis, bullae, synechiae) can result in long-term adhesions or stenosis and may lead to urinary retention. Ocular involvement occurs in 60-100% of cases and may manifest as conjunctival redness, pseudomembranes, or complete corneal epithelial defects. Acute eye findings are the strongest predictor of long-term complications, which can include eyelid malpositions (ectropion/entropion), abnormal lash growth, symblepharon, persistent corneal issues, dry eyes, and reduced vision [12].

Acute kidney injury (AKI) is seen in 20-30% of cases, with proteinuria in up to 60% of patients. In one study, about 10% required renal replacement therapy, and these patients had much higher in-hospital mortality (82% vs. 9%), especially when severe renal failure, large BSA involvement, and high Severity-of-Illness Score for Toxic Epidermal Necrolysis (SCORTEN) scores were present [13]. Respiratory complications are common during the acute phase. These can range from specific lung injuries (such as bronchial epithelial sloughing) to broader issues like pneumonia, pulmonary edema, and atelectasis, with approximately 25% of affected patients developing respiratory failure requiring mechanical ventilation. Gastrointestinal involvement, though less defined, may present with abdominal pain, diarrhea, gastrointestinal bleeding, or ileus. In one intensive care unit study, 55% of patients undergoing endoscopy showed SJS/TEN-related lesions, mostly in the esophagus [14]. Drug-induced liver injury is reported in 13-30% of cases, with risks increased by underlying liver disease, hyperlipidemia, or diabetes.

Hematologic abnormalities, including anemia, leukopenia (observed in about 13% of cases), and thrombocytopenia, are frequent, with disseminated intravascular coagulation occurring in over 20% [15]. Additionally, bacteremia is present in 30-50% of patients, increasing mortality risk three- to fourfold. Sepsis and septic shock account for roughly half of all SJS/TEN-related deaths, most commonly due to pathogens like *Staphylococcus aureus*, *Pseudomonas aeruginosa*, and other gram-negative bacteria. 

In this case, a 76-year-old lady referred from the General Practitioner (GP) presented with a history of spreading itchy, flaking, hyperkeratotic skin rash covering more than 80% of BSA, which started as a rash around her lower abdomen two days back. She was started on two courses of flucloxacillin and doxycycline with a topical steroid from the GP. Following this, the rash became widespread. The drug was stopped immediately, and the patient was referred to us for further management. There is no previous history of drug allergy. Patient is a known case of Sjögren’s, rheumatoid arthritis (RA), chronic obstructive pulmonary disease (COPD), and osteoarthritis (OA).

Pre-admission and during the course of treatment, there was no temperature spike, but after two days of admission, the patient became hypothermic with a temperature of 34.4°C, and then throughout the course, it was 35-36°C.

On arrival, physical examination showed flaky eruptive lesions seen covering more than 80% BSA (covering face, abdomen, torso, and back) with a positive Nikolsky sign. Eyes, lips, and mouth, vulval mucosa were not involved. The patient was reviewed by both ENT and Ophthalmology - ruled out eye/oromucosal involvement.

**Figure 1:**
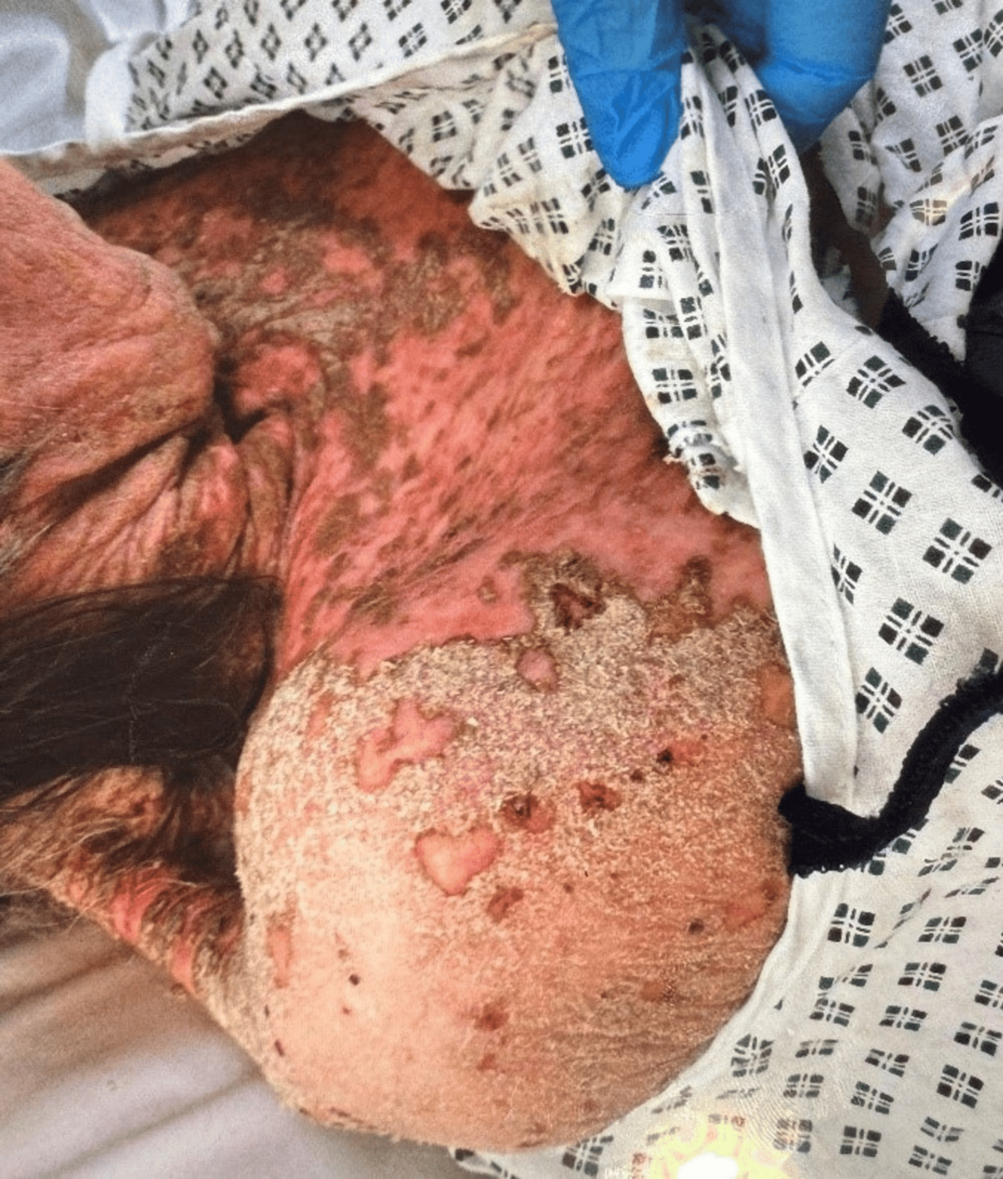
Extensive skin involvement on the patient's back and shoulder, characterized by diffuse erythema, significant scaling, and areas of thick, yellowish-brown crusting. Note the prominent desquamation and areas of skin breakdown.

The image shows an extensive erythema (redness) and desquamation (peeling or flaking of the skin), prominent in the lower part of the area, along with silvery-white scales that are apparent. There are also areas of yellowish-brown crusting, showing exudation or secondary infection, and some scattered erosions or excoriations. The skin appears thickened and fissured in some areas, indicative of chronic inflammation (Figure 1).

**Figure 2:**
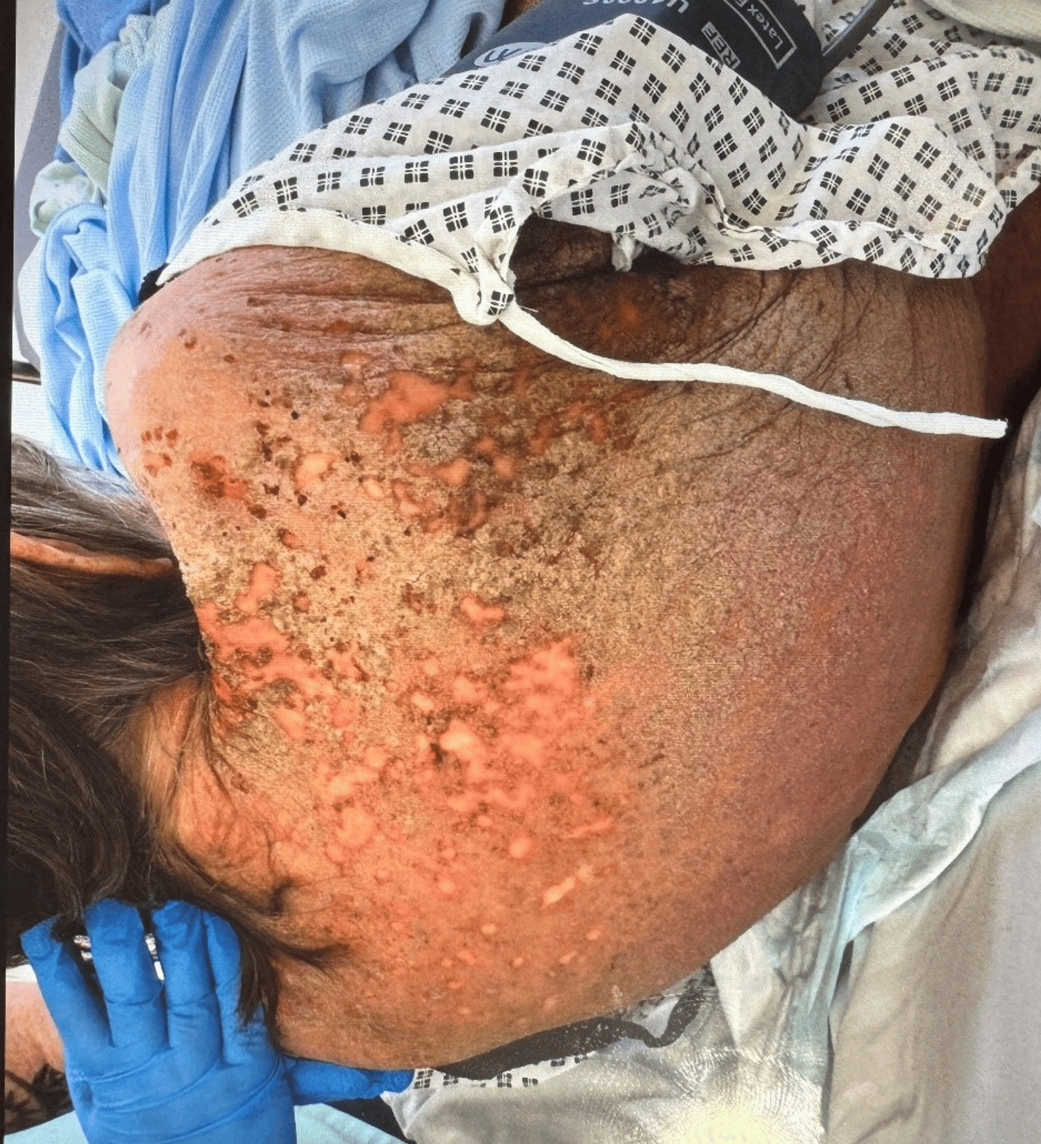
Widespread erythematous and excoriated lesions on the patient's back and flank, with areas of significant crusting and possible serous discharge, consistent with a severe inflammatory dermatosis.

The figure shows the affected skin portion of the back and flank. The skin exhibits widespread erythema (redness) and numerous excoriations with areas of crusting and possibly serous or hemorrhagic discharge, along with visible areas of hyperpigmentation and hypopigmentation, suggesting chronic changes. There is also the presence of widespread erythema, crusting, and a severe inflammatory process and secondary infection (Figure 2).

**Figure 3:**
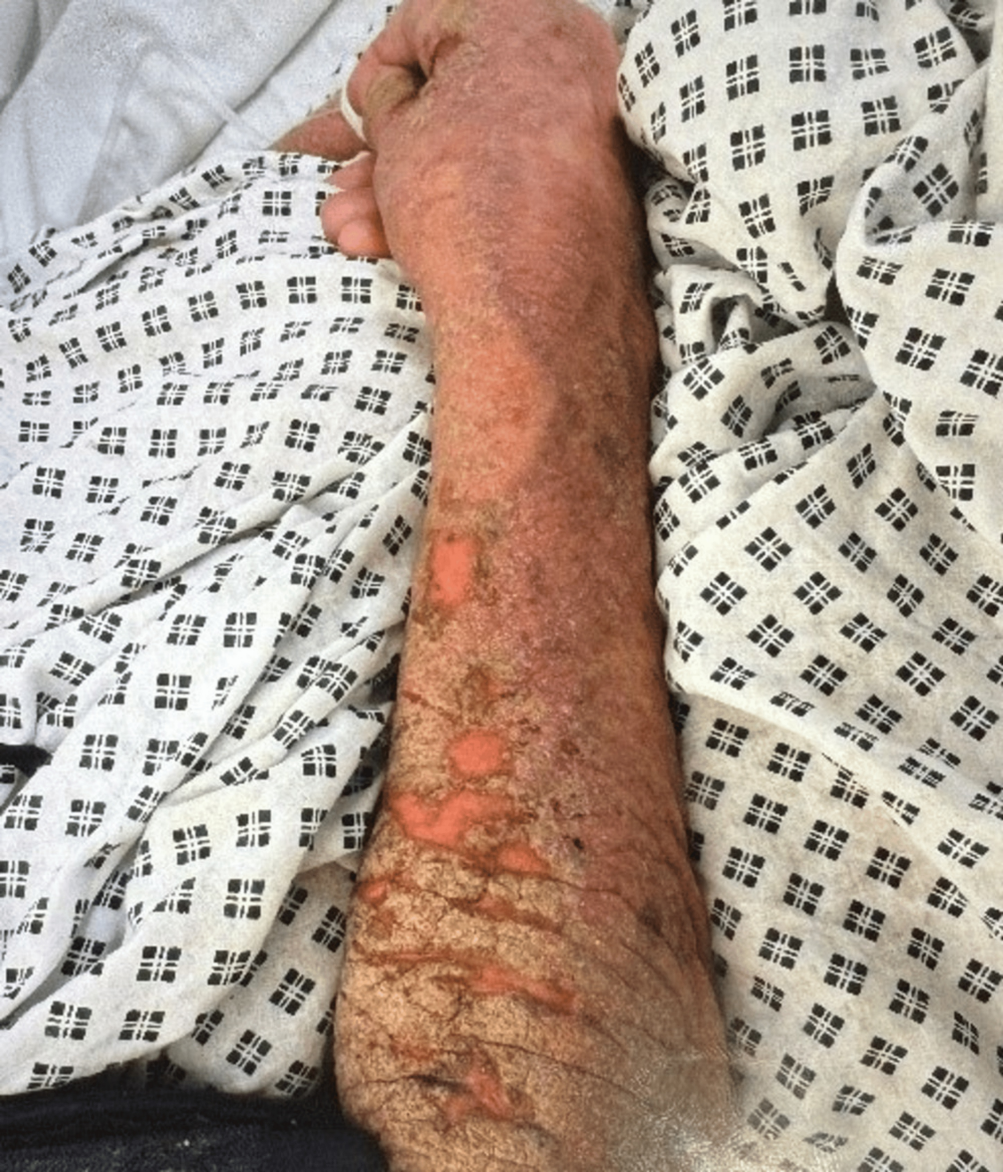
Clinical presentation of the patient's forearm and hand showing extensive erythema, desquamation with fine and coarse scaling, and fissuring. Discrete erythematous patches with some possible vesiculation are also noted on the forearm.

The skin appears intensely reddened (erythematous) and shows significant peeling (desquamation), accompanied by pronounced scaling and fissuring, especially over the forearm and wrist. The scales are predominantly fine and dry, though some regions exhibit a yellowish or brownish hue, likely due to crusting or dried exudate. Additionally, a few distinct, well-defined erythematous patches with a slightly elevated or vesicular texture stand out against the overall inflamed and scaly backdrop (Figure 3).

On evaluation, the patient had a SCORTEN score of four, which carries a 50% mortality risk.

Investigations

Skin biopsy was taken, which showed extensive epidermal necrosis with Immunohistochemistry confirming the diagnosis. The patient was diagnosed to have TEN, with doxycycline/flucloxacillin as the trigger factor (SCORTEN score: 4). Daily skin swabs and routine investigation were performed to evaluate for secondary infections. Intensive care management was recommended, with improvement in the SCORTEN score.

On admission, blood investigations showed hemoglobin of 98 g/L, white blood cell count of 7.5 × 10⁹/L, platelet count of 103 × 10⁹/L, sodium 142 mmol/L, potassium 4.6 mmol/L, urea 25.2 mmol/L, creatinine 112 µmol/L, and an estimated glomerular filtration rate (eGFR) of 41 mL/minute/1.73m², indicating AKI stage 1. Liver function tests revealed total bilirubin of 4 µmol/L and ALT of 68 IU/L. Inflammatory markers were elevated, with CRP at 182 mg/L and ESR at 39 mm/hour. Two days post-admission, hemoglobin dropped to 82 g/L, WBC to 2.8 × 10⁹/L, and platelets to 36 × 10⁹/L. Blood cultures showed no growth, but a skin swab was positive for *Pseudomonas aeruginosa*.

On admission, the patient was started on IV amoxicillin (one dose), which was later changed to IV teicoplanin (four doses) following a microbiology discussion. Supportive measures included IV fluids, liquid paraffin, silver sulfadiazine, and pain management. The patient received IV methylprednisolone (120 mg in 250 mL normal saline) for three days. The room temperature was maintained between 25-32°C to address hypothermia, and the patient was supported with an air mattress and a high-calorie diet. Additionally, intravenous immunoglobulin (IVIG) was administered at 2 g/kg over five days (approximately 18 g/day for a 45-kg patient). Inpatient evaluations by ENT and Ophthalmology led to further supportive treatment recommendations.

In this case, as the age of the patient is 77 and the SCORTEN score is four, indicative of more than 50% mortality, the decision was made to provide supportive care. Unfortunately, the patient succumbed to death on post-admission day despite the best treatment modality.

## Discussion

Differential diagnosis includes EM, which is an immune-mediated condition most often triggered by infections and marked by classic target lesions: circular papules or plaques with a dark, sometimes blistering center, a pale pink middle ring, and a bright red outer border. EM typically affects the extremities with limited involvement. In contrast, SJS/TEN features atypical, flat target lesions without the three-ring pattern, primarily affecting the trunk and head/neck, along with dusky erythema, flaccid bullae, and widespread skin detachment. Histologically, EM shows an interface dermatitis with a lichenoid infiltrate and basal layer necrosis, whereas SJS/TEN presents with extensive epidermal necrosis and minimal inflammation [16].

Exanthematous drug eruptions often present as a generalized, symmetric maculopapular erythema similar to early SJS/TEN, but they lack mucosal involvement, severe skin pain, blisters, and epidermal detachment. Drug reaction with eosinophilia and systemic symptoms (DRESS) presents with a widespread, polymorphic rash, ranging from exanthematous eruptions and confluent erythema to generalized exfoliative dermatitis, without sheet-like detachment.

Severe AGEP can resemble SJS/TEN. It typically develops a few days after exposure to a drug, most often a beta-lactam antibiotic, and presents with numerous pinpoint pustules on an erythematous background. Its defining histologic feature is a spongiform subcorneal and/or intraepidermal pustule. Staphylococcal scalded skin syndrome (SSSS) primarily affects children but can also occur in immunocompromised adults or those with renal disease. Reactive infectious mucocutaneous eruption (RIME) is a parainfectious mucocutaneous reaction, primarily linked to *Mycoplasma*, but also associated with *Chlamydia pneumoniae*, human metapneumovirus, SARS-CoV-2, parainfluenza, and influenza B. It presents as prominent mucositis affecting multiple mucosal sites, with minimal or absent skin involvement.

Outcome and prognosis

Mortality in SJS/TEN averages 25%, with SJS at 10% and TEN exceeding 30%. US data (2009-2012) report 4.8% for SJS, 19.4% for SJS/TEN, and 14.8% for TEN. Pediatric mortality is significantly lower (0-9.5%) but can reach 39% in infants with TEN.

Fatal outcomes often result from sepsis, acute respiratory distress syndrome (ARDS), disseminated intravascular coagulation (DIC), and multiple organ failure, with increased risk in elderly patients (>70 years), those with acute renal failure requiring replacement therapy, cirrhosis, or metastatic cancer. While skin involvement is the primary predictor for 90-day mortality, risk persists. European data show a 23% mortality rate at six weeks and a 34% rate at one year [17].

Recurrence of SJS/TEN in adults is rare, typically seen only with re-exposure to the same or similar drugs. While ICD-based studies report recurrence rates of up to 7% in adults (16 episodes per 1000 person-years) and 18% in children, a validated cohort study showed a lower rate of 4.2 episodes per 1000 person-years. These differences may stem from variable coding practices that can inadvertently include other recurrent conditions, such as EM or bullous fixed drug eruptions. In children, nearly 50% of cases are attributed to infections (e.g., *Mycoplasma*) or remain idiopathic, which may explain the higher observed recurrence rate.

Long-term sequelae from SJS/TEN are varied and affect multiple systems as survival improves. Up to 80% of survivors suffer cutaneous complications, including pigment changes, scarring, eruptive nevi, telogen effluvium, chronic pruritus, and nail abnormalities like Beau lines or onychomadesis. Ocular sequelae - present in 50-90% of patients with acute eye involvement - range from dry eyes and photophobia to corneal scarring and, rarely, blindness. Oral and dental issues may include discomfort, xerostomia, gingival inflammation, and, in children, significant dental developmental disturbances. Genital complications such as adhesions, stenosis, and, in females, introital narrowing, can occur in a notable proportion of survivors. Pulmonary issues, including chronic bronchitis, bronchiolitis, and diminished lung diffusion capacity, are also common. Additionally, approximately one-third of patients experience chronic pain, while many face long-term psychological challenges such as depression, anxiety, and post-traumatic stress, significantly affecting quality of life and work capacity. In this case, as the age of the patient is 77 and the SCORTEN score is four, indicative of more than 50% mortality, the decision was made to provide supportive care. Unfortunately, the patient succumbed to death on post-admission day despite the best treatment modality.

Doxycycline hyclate is a water-soluble tetracycline antibiotic with broad-spectrum activity against gram-positive and gram-negative bacteria. It treats infections such as acne, malaria, skin infections, STDs (including chlamydia, syphilis, gonorrhea, and PID), Lyme disease, and outbreaks like cholera, mycoplasma, tularemia, typhus, and *Rickettsia*. Beyond its antimicrobial role, it offers immunomodulatory and anti-inflammatory benefits useful in RA, various skin disorders (e.g., acne vulgaris, rosacea), and adult periodontal disease via anti-collagenase and anti-matrix metalloproteinase actions. Its high lipophilicity ensures efficient systemic distribution, entering gram-negative bacteria via OmpF/C porins as a cationic complex and crossing gram-positive membranes by an energy-dependent proton motive force. Doxycycline is bacteriostatic, binding the 30S ribosome to block the EF-Tu/GTP/aminoacyl-tRNA complex from the A site, thereby halting protein synthesis. Resistance arises from ribosomal protection proteins (Tet(O) and Tet(M)) that dislodge the drug, permitting protein synthesis to resume [18].

Researchers have reported rare but serious adverse reactions to doxycycline. One case involved a type I anaphylactic reaction - hypotension, bronchospasms, and urticaria - in a patient given IV doxycycline with a beta-blocker during anesthesia [19]. Another case noted that oral doxycycline led to fever, lymphadenopathy, nephritis, hepatitis, and severe pneumonitis with respiratory failure. Rarely, doxycycline hyclate has triggered SJS, characterized by widespread purpuric or targetoid lesions that require hospital care (e.g., hydroxyzine, mupirocin, prednisone). Also, its use with warfarin may enhance anticoagulation through albumin-binding competition and cytochrome P450 inhibition. Additionally, doxycycline can cause hepatic injury, hepatocellular, cholestatic, or mixed, typically appearing one to two weeks after starting therapy, sometimes with DRESS syndrome features; liver biopsies have revealed centrilobular necrosis or cholestasis, with recovery generally occurring within four to six weeks after discontinuation and corticosteroid treatment.

Another case study reported depicted a rare case of doxycycline-induced SJS in a 46-year-old man [20].

Flucloxacillin, a penicillin beta-lactam antibiotic, primarily treats infections caused by gram-positive bacteria but also shows activity against some gram-negative organisms. It inhibits bacterial cell wall synthesis by targeting penicillin-binding proteins (PBPs), leading to cell lysis. Its resistance to beta-lactamases, including penicillinases and extended-spectrum beta-lactamases, enhances its efficacy.

Beta-lactams often cause skin reactions, with morbilliform rash being the most common. EM presents with target lesions, progressing to SJS if the mucosa is involved. Exfoliative dermatitis causes widespread erythema and scaling, while TEN is marked by epidermal detachment and a positive Nikolsky sign. Hypersensitivity angiitis presents as palpable purpura, and photosensitivity reactions are also possible.

Maculopapular rashes occur in 5-10% of patients, especially children with viral infections. They develop within one to two weeks of therapy but may appear earlier in sensitized individuals or later in rare cases. These rashes resolve within seven to 14 days of stopping the antibiotic, despite initial symptom worsening.

Penicillin tolerance in adults after delayed cutaneous reactions is poorly studied. A study involving 642 patients found skin testing had limited predictive value. Mild immediate reactions occurred in 1.5% and day 1 reactions in 4%, with no severe outcomes, highlighting the need for larger studies.

TEN is the most severe form of drug-induced skin reactions, characterized by widespread skin detachment and necrosis affecting over 30% of the BSA. It is an uncommon, immune-mediated mucocutaneous reaction. High-risk drugs include antiepileptics, such as phenytoin, carbamazepine, lamotrigine, allopurinol, and oxicam NSAIDs. Antibiotics like sulfa drugs and aminopenicillins are also frequent culprits. In contrast, tetracyclines are rarely linked to TEN.

A review of the literature shows very few cases of doxycycline-induced SJS (a variant of TEN with under 10% skin involvement) and even fewer of full-blown TEN. A PubMed search from 1974 to 2023 identified only three reports. For instance, Adelman et al. described a 39-year-old woman who developed a blistering rash with 50% body involvement two weeks after a week-long doxycycline course [21]. Similarly, Kulkarni et al. documented an 85-year-old woman who developed TEN with 40% skin sloughing and a positive Nikolsky sign after re-exposure to doxycycline eight months later [22]. Kuster et al. reported a case in a 33-year-old woman with lupus who experienced onychomadesis, painful neuropathy in the hands, and severe chronic keratitis and conjunctivitis following doxycycline-induced TEN [23].

SCORTEN is a widely used TEN severity score based on seven factors: age ≥40, heart rate ≥120/minute, history of cancer/hematologic malignancies, >10% body surface involvement, serum urea >10 mmol/L, serum bicarbonate <20 mmol/L, and serum glucose >14 mmol/L. However, its accuracy has been questioned. For instance, in a retrospective study of 24 TEN patients, SCORTEN showed 100% sensitivity but only 23.81% specificity, overestimating mortality (41.9% predicted vs. 12.5% observed), potentially due to effective treatments like plasmapheresis and IVIG. Additionally, a meta-analysis by Torres-Navarro et al. found that treatments such as cyclosporine or IVIG, combined with corticosteroids, were associated with lower mortality than SCORTEN predicts [24].

Table 1 shows SCORTEN score criteria.

**Table 1:** Severity-of-Illness Score for Toxic Epidermal Necrolysis (SCORTEN) score

Risk factor	0	1
Age	<40 years	>40 years
Associated malignancy	No	Yes
Heart rate (beats/minute)	<120	>120
Serum blood urea nitrogen (BUN) (mg/dL)	<28	>28
Detached body surface	<10%	>10%
Serum bicarbonate (mEq/L)	>20	<20
Serum glucose (mg/dL)	<252	>252

## Conclusions

SJS is a potentially fatal, drug-induced reaction affecting the skin and mucous membranes. We report a rare case of SJS/TEN caused by doxycycline and flucloxacillin in a 77-year-old woman with multiple comorbidities (Sjögren’s syndrome, RA, COPD, and OA). Initially, she developed an itchy, hyperkeratotic rash on her lower abdomen that rapidly spread to over 80% of her body after two courses of these antibiotics alongside topical steroids. Physical examination revealed flaky lesions with a positive Nikolsky sign, and a skin biopsy confirmed extensive epidermal necrosis with immunohistochemistry supporting a TEN diagnosis (SCORTEN 4). Despite aggressive treatment with IV teicoplanin, clindamycin, a three-day course of IV methylprednisolone followed by IVIG, and comprehensive supportive care, her condition worsened, and she ultimately died. Although early recognition and immediate withdrawal of the offending agent is the mainstay of SJS management, our patient’s condition worsened despite the timely implementation of these interventions.
